# Quantifying the effects of ecological constraints on trait expression using novel trait‐gradient analysis parameters

**DOI:** 10.1002/ece3.3541

**Published:** 2017-11-30

**Authors:** Gianluigi Ottaviani, James L. Tsakalos, Gunnar Keppel, Ladislav Mucina

**Affiliations:** ^1^ School of Biological Sciences The University of Western Australia Perth WA Australia; ^2^ Institute of Botany Academy of Sciences of the Czech Republic Třeboň Czech Republic; ^3^ School of Natural and Built Environments and Future Industries Institute Adelaide SA Australia; ^4^ Department of Geography and Environmental Studies Stellenbosch University Stellenbosch South Africa

**Keywords:** bark thickness, biotic interactions, ecological forces, environmental filters, functional trait space, gradient analysis, trait‐based community ecology

## Abstract

Complex processes related to biotic and abiotic forces can impose limitations to assembly and composition of plant communities. Quantifying the effects of these constraints on plant functional traits across environmental gradients, and among communities, remains challenging. We define ecological constraint (*C*_*i*_) as the combined, limiting effect of biotic interactions and environmental filtering on trait expression (i.e., the mean value and range of functional traits). Here, we propose a set of novel parameters to quantify this constraint by extending the trait‐gradient analysis (TGA) methodology. The key parameter is ecological constraint, which is dimensionless and can be measured at various scales, for example, on population and community levels. It facilitates comparing the effects of ecological constraints on trait expressions across environmental gradients, as well as within and among communities. We illustrate the implementation of the proposed parameters using the bark thickness of 14 woody species along an aridity gradient on granite outcrops in southwestern Australia. We found a positive correlation between increasing environmental stress and strength of ecological constraint on bark thickness expression. Also, plants from more stressful habitats (shrublands on shallow soils and in sun‐exposed locations) displayed higher ecological constraint for bark thickness than plants in more benign habitats (woodlands on deep soils and in sheltered locations). The relative ease of calculation and dimensionless nature of *C*_*i*_ allow it to be readily implemented at various scales and make it widely applicable. It therefore has the potential to advance the mechanistic understanding of the ecological processes shaping trait expression. Some future applications of the new parameters could be investigating the patterns of ecological constraints (1) among communities from different regions, (2) on different traits across similar environmental gradients, and (3) for the same trait across different gradient types.

## 1 INTRODUCTION

A central goal of community ecology is to understand the assembly processes shaping biotic communities (Diamond, 1975; Kraft et al., 2015; Mayfield & Levine, 2010; Weiher & Keddy, 1995). Both stochastic (Hubbell, 2001; Ricklefs, 2008) and deterministic (Cornwell & Ackerly, 2009; Maire et al., 2012; Silvertown, 2004) mechanisms operate in structuring plant communities (Cornwell & Ackerly, 2009; Gross et al., 2013; Spasojevic & Suding, 2012). Various pressures, such as resource stress, disturbance regime, and biotic interactions can either promote or prevent species coexistence, affecting the composition and size of species pools (Baraloto et al., 2012; Belyea & Lancaster, 1999; Houseman & Gross, 2006).

Environmental filtering (Cornwell, Schwilk, & Ackerly, 2006; Freschet et al., 2011; Fukami, Bezemer, Mortimer, & van der Putten, 2005) and limiting similarity have traditionally been considered to be the predominant ecological processes in community assembly (MacArthur & Levins, 1967; Pacala & Tilman, 1994; Stubbs & Wilson, 2004). The former is mainly associated with plant–environment relationships, that is, the ecological response of plants to prevalent environmental conditions (Cornwell et al., 2006; Kraft et al., 2015; Maire et al., 2012). Limiting similarity is, on the other hand, more related to the role of biotic interactions among species in a plant community, such as competition (Schwilk & Ackerly, 2005; Silvertown, 2004; Stubbs & Wilson, 2004).

In addition, other ecological mechanisms are increasingly considered important in structuring community assembly. Facilitation (Armas, Schöb, & Gutíerrez, 2013; McIntire & Fajardo, 2014; Schöb et al., 2014), the effects of pathogens (Albornoz, Burgess, Lambers, Etchells, & Laliberté, 2017), and parasitism (Schöb et al., 2014) have been shown to be influential in this context. Furthermore, intraspecific trait variability is also important in driving community assembly (Bolnick et al., 2011; de Bello et al., 2011; Jung, Violle, Mondy, Hoffmann, & Muller, 2010; Siefert et al., 2015; Violle et al., 2012), along with trait differences among species (Auger & Shipley, 2013; Kichenin, Wardle, Peltzer, Morse, & Freschet, 2013; Kraft, Crutsinger, Forrestel, & Emery, 2014; Kraft et al., 2015). It is therefore crucial to consider the combined effects of all these mechanisms operating simultaneously during community assembly (Gross et al., 2013; Maire et al., 2012; Spasojevic & Suding, 2012).

The aforementioned ecological processes can impose constraints to (1) species occurrence (de Bello et al., 2012; Götzenberger et al. 2012), (2) trait expression (trait mean values and ranges; Albert et al., 2010), (3) trait combinations and covariance (Díaz et al., 2016; Dwyer & Laughlin, 2017a), and (4) trait diversity (Bernard‐Verdier et al., 2012; Butterfield & Suding, 2013; Spasojevic & Suding, 2012). Assessing the magnitude of these constraints on species niches, plant traits, and community assembly across environmental gradients and at various scales (e.g., from population to biome) remains challenging (Dwyer & Laughlin, 2017a,2017b). Therefore, methods that can quantify the effects of biotic and abiotic constraints on the expression and diversity of traits are needed to better understand community assembly processes.

The trait‐gradient analysis (TGA; Ackerly & Cornwell, 2007) links single traits to environmental gradients and allows quantifying within‐ and among‐community components of species’ trait parameters. TGA illustrates the responses of traits in species along environmental gradients, but it does not allow quantifying the effects of ecological constraints imposed by key environmental parameters on trait expression along these gradients. In this study, we use the theoretical and methodological framework of TGA to develop new parameters that can quantify these effects on trait expression. We then illustrate the utility of the main novel parameter, ecological constraint (*C*_*i*_), by demonstrating its ability to explain changes in bark thickness of dominant woody species across granite outcrops in southwestern Australia.

## 2 THE NEW TGA PARAMETERS

The species niche is an essential concept in ecology (e.g., Hutchinson, 1957; Whittaker, 1960) and its quantification has remained challenging, although some methods have been proposed (e.g., Laughlin & Joshi, 2015; Mason, de Bello, Doležal, & Lepš, 2011; Urbina et al., 2017). Functional traits are considered crucial elements for identifying a species’ niche in a given habitat, community, or area (Ackerly & Cornwell, 2007; Butterfield, Bradford, Munson, & Gremer, 2017; Mason et al., 2011). TGA has facilitated the quantification of the functional aspect of species niches and the assessment of niche breadth (Ackerly & Cornwell, 2007). Here, we extend this approach to enable quantifying the effects of ecological constraint on trait expression.

**Figure 1:**
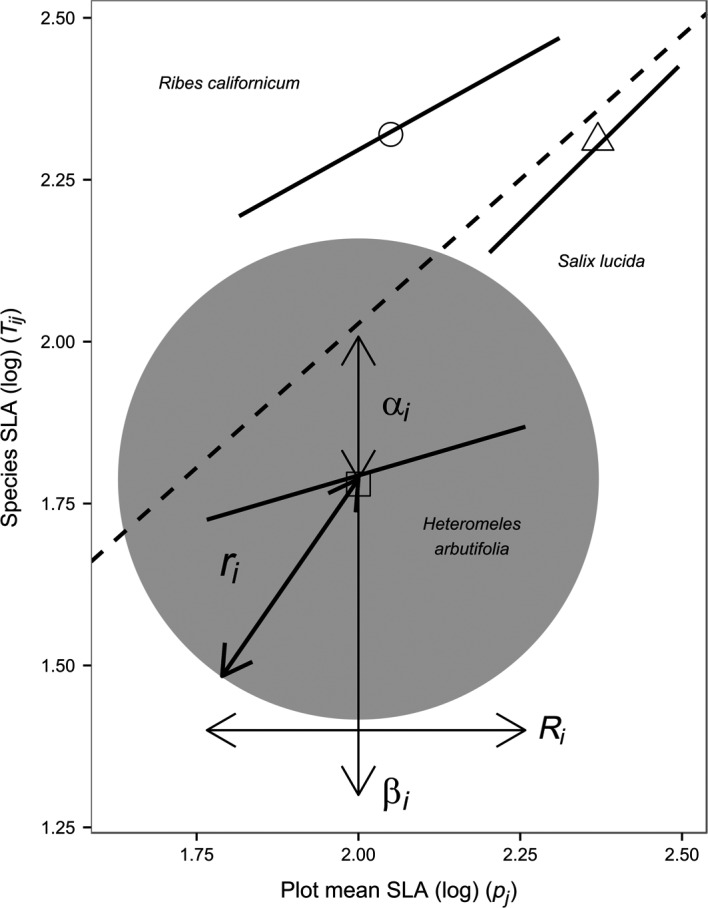
SLA trait gradient for three species of Californian chaparral (Jasper Ridge Biological Preserve). The dashed line indicates the trait community average (X = Y). The proposed TGA parameters are plotted, as an example, for *Heteromeles arbutifolia*: functional trait niche space (FTNS_*i*_, outlined by the gray circle) and trait range (*r*_*i*_, the radius of FTNS_*i*_ indicative of the species *S*_*i*_ trait range). The original TGA parameters alpha (α_*i*_), beta (β_*i*_), and niche breadth (*R*_*i*_) parameters are also reported (modified from Ackerly & Cornwell, 2007)

Trait‐gradient analysis plots plant communities along a two‐dimensional trait‐space gradient. The trait values of species within a plot (*y*‐axis) are plotted against their trait values across communities on the *x*‐axis. TGA therefore partitions the mean trait values for an individual species into within‐site (alpha, α_*i*_) and among‐site (beta, β_*i*_) components (Figure 1; Table 1). Beta estimates the species’ mean position along the trait gradient as the projection on the *x*‐axis of the mid‐point of the species regression line (derived from trait values in plots where the species S_i_ occurs along the environmental gradient). The alpha component is calculated as the difference between the mean trait value at a site of locally, co‐occurring taxa and its beta value, hence indicating how a species’ mean trait value at a site differs from that of all other co‐occurring species (Figure 1; Table 1). Therefore, we can infer that alpha is more affected by within‐site biotic interactions, whereas beta is more determined by among‐site abiotic drivers (Ackerly & Cornwell, 2007; Cornwell & Ackerly, 2009).

**Table 1:** Definition and characteristics of key TGA components from the original framework by Ackerly and Cornwell (2007) and this study

TGA parameters	Definition, calculation, dimensionality, and ecological meaning
From Ackerly and Cornwell (2007)	
Alpha (α_*i*_)	Average distance of the species *S*_*i*_ from the co‐occurring species in the study system, for example, community. It is measured along the *y*‐axis as the difference between the mid‐point of the species regression line and the trait community average. One‐dimensional. It mainly relates to biotic interactions.
Beta (β_*i*_)	Species *S*_*i*_ mean location along a trait‐environment gradient. It is calculated as the projection of the species mid‐point regression line on the *x*‐axis. One‐dimensional. It indicates the effect of abiotic factors on the average plant placement across the gradient.
Niche breadth (*R*_*i*_)	Trait span across communities in the study system. It is calculated as the projection on the *x*‐axis of the entire length of the species regression line, as inferred from plots in which the species *S*_*i*_ occurs along the gradient. One‐dimensional. It provides insights on the overall variability of a species trait values across a trait‐environment gradient.
This study	
Functional trait niche space (FTNS_*i*_)	Average functional space a species can occupy in a given system across a trait‐environment gradient. It is calculated as the product of the biotic‐related α_*i*_, and the abiotic‐related β_*i*_, assuming a circular area (associated with normality of data distribution) around the species *S*_*i*_ mid‐point regression line. Two‐dimensional. This parameter considers both biotic and abiotic forces in shaping the average niche space of a species trait.
Trait range (*r*_*i*_)	Average trait variability the species *S*_*i*_ can span across a trait‐environment gradient. It is calculated as the FTNS_*i*_ radius. One‐dimensional. It represents a proxy for the average trait (and niche) space a species can occupy in a given system.
Ecological constraints (*C*_*i*_)	Average effect of ecological forces on a species’ trait expression. It is calculated as the ratio between the β_*i*_ and *r*_*i*_ values. Dimensionless. It quantifies the magnitude of the biotic and abiotic constraints imposed on trait average and variability.

Ackerly and Cornwell (2007) proposed niche breadth (*R*_*i*_) to be the one‐dimensional projection of the species regression line on the *x*‐axis (Figure 1; Table 1). Hence, the niche breadth is related to the position and range occupied by the species along the trait‐environment gradient (Ackerly & Cornwell, 2007). We here propose a new, complementary two‐dimensional parameter, the functional trait niche space (FTNS_*i*_; Equation (1)). This new measure is the product of alpha (more related to biotic interactions) and beta (more associated with environmental pressures) trait values of the species *S*_*i*_ (Figure 1; Table 1). We suggest that FTNS_*i*_ quantifies the overall role of different abiotic and biotic factors in shaping the niche space occupied by a species.

(1) $${\text{FTNS}}_{i}=\left|\alpha_{i}\beta_{i}\right|$$

Because α_*i*_ and β_*i*_ can assume both positive and negative values, we use the absolute value of their product to calculate FTNS_*i*_. FTNS_*i*_ represents the average two‐dimensional single functional trait space that the species *S*_*i*_ can occupy along a trait gradient. It can be interpreted as the average potential trait space of a species (Figure 1; Table 1). We consider the functional trait niche space to be circular, based on the assumption of normally distributed data. Note that significant departure from this assumption would invalidate the calculation of further parameters. We propose that FTNS_*i*_ is centered around the mid‐position of the species regression line, that is, from where β_*i*_ is then inferred by its projection on the *x*‐axis, indicating the average niche location of a species *S*_*i*_ along the trait gradient. Hence, the radius (*r*_*i*_; Equation (2)) of FTNS_*i*_ can be derived as:

(2) $$r_{i}={\left({\text{FTNS}}_{i}/\pi \right)}^{-2}$$

*r*_*i*_ represents the mean trait range of the species *S*_*i*_ and has the same unit of measurement as α_*i*_ and β_*i*_ parameters. We suggest that *r*_*i*_ is related to both biotic and abiotic factors and quantifies the average single‐trait range of species *S*_*i*_ in the studied system, as it represents the one‐dimensional measure (i.e., the radius) of the two‐dimensional FTNS_*i*_ (Figure 1; Table 1). We further propose a parameter for ecological constraints (*C*_*i*_) on a single trait of a single species, as the ratio between a species’ mean location along the trait‐environment gradient, β_*i*_, and its mean trait range, *r*_*i*_ (Equation (3); Table 1):

(3) $$C_{i}=\beta_{i}/r_{i}$$

We propose that *C*_*i*_ estimates the average impact of the ecological constraints on trait expression at the species level. Beta is related to ecological constraint because it indicates a species’ mean location along the trait‐environment gradient, which is strongly affected by abiotic pressures. Ecological constraints should also impact the species’ trait range, *r*_*i*_, as greater constraints would reduce the range, that is, producing smaller ranges due to smaller niche space. Therefore, the ratio between the beta position and the trait range is sensitive to the combined effects of ecological constraints on both the average species position along the trait‐environment gradient and on species trait range. *C*_*i*_ should therefore provide a good indication of the average effect of limitations on trait expression imposed by biotic and abiotic forces. Notably, the ecological constraint is dimensionless, a property allowing the quantification (and the comparison) of the impact of *C*_*i*_ at different scales and across different environmental gradients.

## 3 IMPLEMENTING THE NEW TGA PARAMETERS: A CASE STUDY

To illustrate the application of the new parameters, we investigated effects of ecological constraint (*C*_*i*_) on the expression of a single trait, bark thickness, for dominant species in shrublands and woodlands on granite outcrops across an aridity gradient in southwestern Australia (Ottaviani, Marcantonio, & Mucina, 2016; Schut et al., 2014). Bark thickness is considered a key plant functional trait, associated with, and responding to, changing fire regime (Pausas, 2017; Rosell, Gleason, Méndez‐Alonzo, Chang, & Westoby, 2014) and climate (Richardson et al., 2015; Rosell, 2016; Rosell et al., 2014). In relation to climatic conditions, plants exhibiting thicker bark are generally found in drier and hotter environments, assisting in the storage of water and photosynthates (Richardson et al., 2015; Rosell, 2016).

The aim of this case study was to provide a first implementation of the novel TGA parameters, particularly *C*_*i*_, and to demonstrate the ecological inferences that these parameters could facilitate. Details about the study area, data collection, and statistical analysis are available in the Appendix S1. We modeled the changes of bark thickness *C*_*i*_ across the aridity gradient. We expected positive relationship between increasing aridity and ecological constraints, as more arid (higher stress) conditions should impose stronger ecological constraints on bark thickness expression than experienced in more mesic (lower stress) sites. We also compared *C*_*i*_ between two vegetation types, shrublands and woodlands. We hypothesized plants occurring in more sun‐exposed, water‐stressed shrubland habitats on shallower soils to experience higher ecological constraints on bark thickness than plants in more sheltered, less water‐stressed woodland habitats on deeper soils (Ottaviani et al., 2016; Schut et al., 2014).

### 3.1 Bark thickness is more ecologically constrained in stressful environments along an aridity gradient

Ecological constraint *C*_*i*_ for bark thickness was strongly and positively correlated with aridity (*t*‐value = 8.65, marginal *R* ^2^ = 0.46 [variance explained by the fixed effect, aridity—see Appendix S1 for details], conditional *R* ^2^ = 0.80, *p *<* *.001). Therefore, plants occurring in more arid environments are more ecologically constrained, which may be due to biotic and/or abiotic factors. This means that plants found in more stressful habitats are converging toward values, with lower variability, of bark thickness closer to the trait community average than plants from more mesic environments. In other words, the ecological constraint imposed by aridity stress (and possibly fire regime; not tested) has selected for a restricted set of values for this trait toward the high‐stress end of the gradient (Richardson et al., 2015).

### 3.2 Bark thickness in shrublands is more ecologically constrained than in woodlands

**Figure 2:**
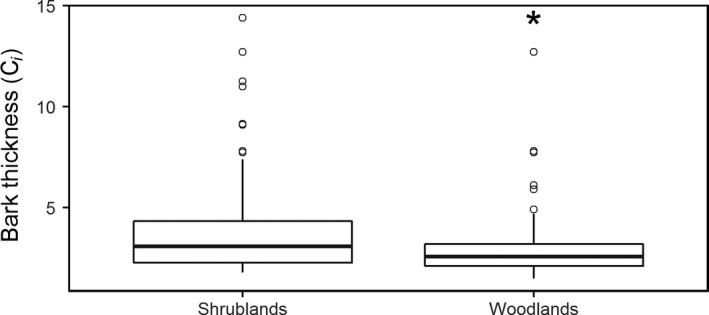
Shrublands and woodlands box and whisker plots of ecological constraint (*C*_*i*_) for bark thickness of dominant woody species on and around southwestern Australian granite outcrops (the asterisk indicates significant difference; *p* < .01)

Bark thickness expression was more constrained in shrublands than in woodlands (Figure 2; Shrublands median *C*_*i*_ = 3.07 ± 0.21 *SE*; Woodlands median *C*_*i*_ = 2.56 ± 0.15 *SE*;* p *<* *.01). Shrublands on granite outcrops are occurring in more stressful conditions (i.e., microhabitats with drier and shallower soils) than woodlands (Schut et al., 2014). This aridity stress is imposing strong limitations on bark thickness expression for plants developing in shrublands, as indicated by lower *C*_*i*_ values in this habitat.

## 4 CONCLUDING REMARKS AND FUTURE DIRECTIONS

We have presented novel TGA parameters for quantifying the role of ecological constraints on trait expression by expanding on the TGA tool box. The power of the proposed TGA tools derives from their relatively simple calculation and the dimensionless nature of *C*_*i*_. Hence, *C*_*i*_ can be implemented at different scales. We have shown that the magnitude of *C*_*i*_ varied across an environmental gradient, and between two different vegetation types, on granite outcrops in southwestern Australia according to predictions based on ecological principles.

In conjunction with the original TGA parameters (in particular alpha, beta, and niche breadth; Ackerly & Cornwell, 2007), the proposed TGA tools (especially *C*_*i*_) could provide a more complete picture of the overall effects of plant–environment relationships and plant–plant interactions on traits. In particular, *C*_*i*_ allows quantifying the impact of ecological constraints on trait expression. Consequently, this set of parameters could assist to better explaining and, potentially, predicting the effects of environmental (e.g., climate) change on plant community assembly and functioning.

Future implementations could explore the variation patterns of ecological constraints for different traits, in different environments and for other plant species and communities. In particular, further studies could focus on (1) comparing *C*_*i*_ among vegetation types from different regions that could display, for example, either different or similar plant trait responses to biotic and abiotic (e.g., climatic, resource availability) drivers, (2) modeling *C*_*i*_ for different traits across similar environmental gradients to investigate which traits are more strongly affected by ecological constraints along particular gradients, and (3) analyzing single‐trait *C*_*i*_ changing pattern across different gradient types to investigate which environmental variables limit the expression of a given trait. Such broader application of the methodology could test its generality, while advancing our understanding of ecological processes determining trait expression and species coexistence in plant communities.

## DATA ACCESSIBILITY

Data available from the Dryad Digital Repository: https://doi:10.5061/dryad.23fg0.

## CONFLICT OF INTEREST

None declared.

## AUTHOR CONTRIBUTIONS

GO conceived the research idea, collected the data, and wrote the first draft of the manuscript. JLT performed the statistical analyses, produced the graphical outputs, and provided comments on the text. GK and LM contributed in developing the original idea and assisted in writing the manuscript.
