# Supplementary material for: Quantifying the effects of ecological constraints on trait expression using novel trait‐gradient analysis parameters
Source: Ecol Evol. 2017 Nov 30;8(1):435–40. doi: 10.1002/ece3.3541 (PMC5756828; doi:10.1002/ece3.3541)

**SUPPLEMENTARY MATERIAL**

**Appendix S1**

**Details of the study area, data collection, statistical analyses related to the case-study.**

STUDY AREA

Southwestern Australia has mediterranean-type climate and is a mega-diverse region, hosting one of the global biodiversity hotspots (Cowling *et al.* 1996; Myers *et al.* 2000). In this ancient, nutrient-poor, fire-prone and flat landscape, numerous dome-shaped granite outcrops occur (Hopper & Gioia 2004). These inselbergs provide habitat diversity and unique microclimates compared to the regional conditions, supporting high species diversity and high levels of endemism (Poot & Lambers 2008; Poot, Hopper & van Diggelen 2012; Keppel *et al.* 2017). The granite outcrops are considered refugia for biota during periods of increasing environmental stress (Keppel *et al.* 2012, 2017; Schut *et al.* 2014). We collected trait data from seven granite outcrops along an aridity gradient, ranging from the mesic SW toward the xeric NE (Ottaviani, Marcantonio & Mucina 2016). The aridity gradient was identified by using six selected, highly informative, bioclimatic variables (from WorldClim; Hijmans et al. 2005) related to temperature and precipitation, namely isothermality, minimum temperature of the coldest month, mean temperature of driest quarter, mean temperature coldest quarter, annual precipitation and precipitation seasonality (for details refer to Ottaviani, Marcantonio & Mucina 2016).

SPECIES SELECTION AND BARK THICKNESS MEASUREMENTS

We collected bark thickness from fourteen woody species (Table I) that were found to be dominant by prior surveys of the vegetation (for details refer to Ottaviani, Marcantonio & Mucina 2016). We investigated two prevalent vegetation types: shrublands and woodlands. Shrublands are composed of two vegetation layers (herbs and shrubs) generally occurring on the slopes, and flattening areas (e.g., tops) of the granite outcrops. Woodlands are characterized by three vegetation layers (defined by herbs, shrubs, and trees) that develop at the edges of inselbergs bordering the surrounding landscapes, where they gain more water and nutrients, compared to shrublands, through run-off from the impermeable granitic slopes.

We gathered bark thickness data from ten healthy, adult individual plants per species per outcrop where the species occurred, targeting individuals of similar age for each species (Pérez-Harguindeguy *et al.* 2013). Each sample came from a different vegetation plot, randomly placed on a granite outcrop. In this way, we could account both for intra- and inter-specific trait variability. For each individual sampled, we averaged the bark thickness from five measurements collected from every stem (average N = 270; Appendix I). We measured bark thickness at breast height for trees and high shrubs (ca. 135 cm above ground; Fajardo & Piper 2011), and at 20 cm above the ground for small shrubs, using a forester penetrating scaled head. We then calculated all TGA parameters for bark thickness for the sampled plant species.

STATISTICAL ANALYSES

We applied linear mixed-effects model (LME; Zuur *et al.* 2009) to test for differences in bark thickness C_i_ across the aridity gradient. LMEs have been suggested to be appropriate models within and across islands and, by extension, insular habitats such as inselbergs (Bunnefeld & Phillimore 2012). The response variable of bark thickness C_i_ (1/log10 transformed to meet normality and homoscedasticity of data distribution) was modelled in relation to aridity (fixed effect). Vegetation and species were set as random factors in these models. Visual inspection of the distribution of residuals did not reveal any deviations from normality and homoscedasticity assumptions. Whilst model validation did not point out immediate problems with homogeneity of variance, the model performance was improved further by allowing for fixed variance per granite outcrop. We obtained the p-value for the full model using a likelihood ratio test against null (intercept-only) model. We then applied the approach proposed by Nakagawa & Schielzeth (2013) to assess the marginal and conditional R^2^ of the LME, which are related to the proportion of variance explained by the fixed factor (in our case aridity) alone and by both fixed and random factors, respectively. We tested for differences in bark thickness (C_i_) between shrubland and woodland communities using the non-parametric Wilcoxon rank sum test with continuity correction, as population means of both groups were not normally distributed. We performed all the analyses in R environment, using combination of TGA script from Ackerly & Cornwell (2007), and R-libraries and packages: *nlme* (Pinheiro *et al.* 2016), *piecewiseSEM* (Lefcheck 2015) and *effects* (Fox 2003).

**References**

Ackerly, D.D. & Cornwell, W.K. (2007) A trait-based approach to community assembly: partitioning of species trait values into within- and among-community components. *Ecology Letters,* **10**, 135–145.

Bunnefeld, N. & Phillimore, A.B. (2012) Island, archipelago and taxon effects: mixed models as a means of dealing with the imperfect design of nature’s experiments. *Ecography,* **35**, 15–22.

Cowling, R.M., Rundel, P.W., Lamont, B.B., Arroyo, M.K. & Arianoutsou, M. (1996) Plant diversity in mediterranean-climate regions. *Trends in Ecology and Evolution,* **11**, 362–366.

Fajardo, A. & Piper, F.I. (2011) Intraspeciﬁc trait variation and covariation in a widespread tree species (Nothofagus pumilio) in southern Chile. *New Phytologist,* **189**, 259–271.

Fox, J. (2003) Effect displays in r for generalised linear models. *Journal of Statistical Software,* **8**, 1–27.

Hijmans, R.J., Cameron, S.E., Parra, J.L., Jones, P.G. & Jarvis, A. (2005) Very high resolution interpolated climate surfaces for global land areas. *International Journal of Climatology*, **25**, 1965–1978.

Hopper, S.D. & Gioia, P. (2004) The Southwest Australian Floristic Region: evolution and conservation of a global hot spot of biodiversity. *Annual Review of Ecology, Evolution, and Systematics,* **35**, 623–650.

Keppel, G., Van Niel, K.P., Wardell-Johnson, G.W., Yates, C.J., Byrne, M., Mucina, L., Schut, A.G.T., Hopper, S.D. & Franklin, S.E. (2012) Refugia: identifying and understanding safe havens for biodiversity under climate change. *Global Ecology and Biogeography,* **21**, 393–404.

Keppel, G., Robinson, T.P., Wardell-Johnson, G.W., Yates, C.J., Van Niel, K.P., Byrne, M. & Schut, A.G.T. (2017) A low-altitude mountain range as an important refugium for two narrow endemics in the Southwest Australian Floristic Region biodiversity hotspot. *Annals of Botany,* **119**, 289–300.

Lefcheck, J.S. (2015) piecewiseSEM: Piecewise structural equation modelling in R for ecology, evolution, and systematics. *Methods in Ecology and Evolution,* **7**, 573–579.

Myers, N., Mittermeier, R.A., Mittermeier, C.G., da Fonseca, G.A.B. & Kent, J. (2000) Biodiversity hotspots for conservation priorities. *Nature,* **403**, 853–858.

Nakagawa, S. & Schielzeth, H. (2013) A general and simple method for obtaining R^2^ from generalized linear mixed-effects models. *Methods in Ecology and Evolution,* **4**, 133–142.

Ottaviani, G., Marcantonio M, Mucina L. 2016. Soil depth shapes plant functional diversity in granite outcrops vegetation of Southwestern Australia. *Plant Ecology & Diversity* 9: 263–276.

Pérez-Harguindeguy, N., Díaz, S., Garnier, E., Lavorel, S., Poorter, H., Jaureguiberry, P., Bret-Harte, M.S., Cornwell, W.K., Craine, J.M., Gurvich, D.E., Urcelay, C., Veneklaas, E.J., Reich, P.B., Poorter, L., Wright, I.J., Ray, P., Enrico, L., Pausas, J.G., de Vos, A.C., Buchmann, N., Funes, G., Quétier, F., Hodgson, J.G., Thompson, K., Morgan, H.D., ter Steege, H., van der Heijden, M.G.A., Sack, L., Blonder, B., Poschlod, P., Vaieretti, M.V., Conti, G., Staver, A.C., Aquino, S. & Cornelissen J.H.C. (2013) New handbook for standardised measurement of plant functional traits worldwide. *Australian Journal of Botany,* **61**, 167–234.

Pinheiro, J., Bates, D., DebRoy, S., Sarkar, D. & R Core Team. (2016) nlme: linear and nonlinear mixed effects models. R package version 3.1-128.

Poot, P. & Lambers, H. (2008) Shallow-soil endemics: adaptive advantages and constraints of a specialized root-system morphology. *New Phytologist,* **178**, 371–381.

Poot, P., Hopper, S.D. & van Diggelen, J.M.H. (2012) Exploring rock ﬁssures: does a specialized root morphology explain endemism on granite outcrops? *Annals of Botany,* **110**, 291–300.

R Core Team. (2016) *R: A language and environment for statistical computing*. R Foundation for Statistical Computing, Vienna.

Schut, A.G.T., Wardell-Johnson, G.W., Yates, C.J., Keppel, G., Baran, I., Franklin, S.E., Hopper, S.D., Van Niel, K.P., Mucina, L. & Byrne, M. (2014) Rapid characterisation of vegetation structure to predict refugia and climate change impacts across a global biodiversity hotspot. *PLoS ONE,* **9**, e82778.

Zuur, A.F., Ieno, E.N., Walker, N.J., Saveliev, A.A. & Smith, G.M. (2009) *Mixed Effects Models and Extensions in Ecology with R*. Springer Science+Business Media, New York.

**Table S1:** The studied dominant woody species used for the bark-thickness measurements. Collection site acronyms: b, Boyagin Rock; kr, Kokerbin Rock; mc, Mount Caroline; mf, Mount Frankland; mtck, Mount Cooke; p, Porongurup; sr, Sandford Rocks (see Ottaviani, Marcantonio & Mucina 2016 for details on location and map of the sites).

| **Species** | **Family** | **Collection site** |
| --- | --- | --- |
| *Acacia lasiocalyx* C.R.P.Andrews | Fabaceae | kr, mc, sr |
| *Acacia* Mill. sp. | Fabaceae | mc |
| *Corymbia calophylla* (Lindl.) K.D.Hill & L.A.S.Johnson | Myrtaceae | b, mtck, p, mf |
| *Dodonaea viscosa* Jacq. | Sapindaceae | sr |
| *Eucalyptus caesia* Benth. | Myrtaceae | b |
| *Eucalyptus loxophleba* Benth. | Myrtaceae | kr, mc |
| *Eucalyptus marginata* Sm. | Myrtaceae | b |
| *Eucalyptus megacarpa* F.Muell. | Myrtaceae | p, mf |
| *Eucalyptus wandoo* Blakely | Myrtaceae | mtck |
| *Eutaxia* sp. | Fabaceae | p, mf |
| *Hakea petiolaris* Meisn. | Proteaceae | mtck, p |
| *Hemiandra* sp. | Lamiaceae | mtck |
| *Kunzea pulchella* (Lindl.) A.S.George | Myrtaceae | kr, mc, sr, b |
| *Trymalium odoratissimum* Lindl. | Rhamnaceae | p, mf |

**Figure S1**. Scatterplot reporting the distribution of C_i_ values in relation to r_i_. The site acronyms follow those in Table I.


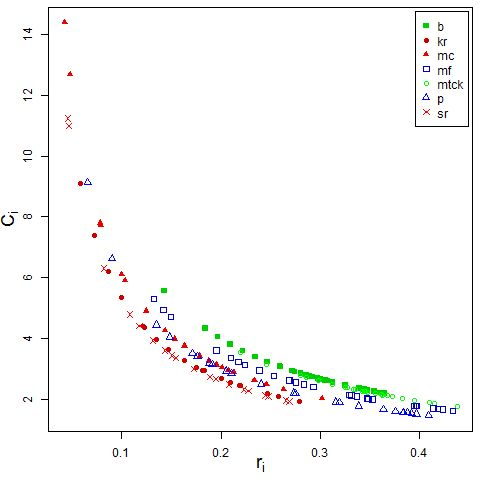

Supplement: Supplementary file 1 [file ECE3-8-435-s001.docx]
